# ANKFN1 plays both protumorigenic and metastatic roles in hepatocellular carcinoma

**DOI:** 10.1038/s41388-022-02380-0

**Published:** 2022-06-21

**Authors:** Yanyan Wang, Yue Zhang, Jiaqi Mi, Chenchen Jiang, Qiang Wang, Xinwei Li, Menglin Zhao, Zhijun Geng, Xue Song, Jing Li, Lugen Zuo, Sitang Ge, Zining Zhang, Hexin Wen, Zishu Wang, Fang Su

**Affiliations:** 1 The First Affiliated Hospital of Bengbu Medical College, Bengbu, 233004 Anhui PR China; 2 Cancer Neurobiology Group, School of Biomedical Sciences & Pharmacy, The University of Newcastle, Callaghan, NSW 2308 Australia; 3 School of Medicine & Public Health, The University of Newcastle, Callaghan, NSW 2308 Australia; 4 Bengbu Medical College, Bengbu, 233004 Anhui PR China

**Keywords:** RNAi, Oncogenes, Experimental organisms, Mechanisms of disease, Gene expression

## Abstract

Ankyrin repeat and fibronectin type III domain containing 1 (ANKFN1) is reported to be involved in human height and developmental abnormalities, but the expression profile and molecular function of ANKFN1 in hepatocellular carcinoma (HCC) remain unknown. This study aimed to evaluate the clinical significance and biological function of ANKFN1 in HCC and investigate whether ANKFN1 can be used for differential diagnosis in HCC. Here, we showed that ANKFN1 was upregulated in 126 tumor tissues compared with adjacent nontumorous tissues in HCC patients. The upregulation of ANKFN1 in HCC was associated with cirrhosis, alpha-fetoprotein (AFP) levels and poor prognosis. Moreover, silencing ANKFN1 expression suppressed HCC cell proliferation, migration, invasion, and metastasis in vitro and subcutaneous tumorigenesis in vivo. However, ANKFN1 overexpression promoted HCC proliferation and metastasis in an orthotopic liver transplantation model and attenuated the above biological effects in HCC cells. ANKFN1 significantly affected HCC cell proliferation by inducing G1/S transition and cell apoptosis. Mechanistically, we demonstrated that ANKFN1 promoted cell proliferation, migration, and invasion via activation of the cyclin D1/Cdk4/Cdk6 pathway by stimulating the MEK1/2-ERK1/2 pathway. Moreover, ANKFN1-induced cell proliferation, migration, and invasion were partially reversed by ERK1/2 inhibitors. Taken together, our results indicate that ANKFN1 promotes HCC cell proliferation and metastasis by activating the MEK1/2-ERK1/2 signaling pathway. Our work also suggests that ANKFN1 is a potential therapeutic target for HCC.

## Introduction

Primary liver cancer is the sixth most commonly diagnosed cancer, ranking fifth in global incidence; it accounted for approximately 906,000 new cancer cases and 830,000 cancer-related deaths and was the third (8.3%) leading cause of cancer-related death worldwide in 2020 [1, 2]. Hepatocellular carcinoma (HCC) is the most common form of primary liver cancer [3]. Chronic hepatitis B virus and aflatoxin are the main causes of liver cancer in China [4, 5], and the high incidence and death rates of liver cancer are still a serious threat to human health in China. Surgical resection is recognized as an effective treatment for early-stage HCC. There are no obvious symptoms in patients with early-stage HCC; for this reason, most patients have already developed advanced cancer at the time of diagnosis and have missed the optimal window for surgical resection or liver transplantation [6, 7]. Despite improvements in screening technology and treatment strategies, recurrence, frequent intrahepatic spread, and extrahepatic metastasis are still the main causes of HCC-related death; the 5-year recurrence rate is as high as 70%, and the median survival of patients is only 6–20 months [8]. Therefore, it is important to improve the overall clinical treatment effect of HCC and reduce its mortality rate by identifying the pivotal molecular markers of HCC and exploring its mechanism.

The ankyrin repeat and fibronectin type III domain containing 1 (ANKFN1) gene is located at 17q22, and studies have shown that patients with microdeletions in this domain present with facial deformities, eyelid ptosis, bilateral index finger deformities, systemic joint stiffness, vertebral abnormalities, and other clinical symptoms. Further molecular analysis revealed that the microdeletion region contains the ANKFN1 gene [9].

ANKFN1 contains both an ankyrin repeat (AR) motif and a fibronectin type III domain (FN3). AR domains were first discovered as a repeating sequence in *Saccharomyces cerevisiae* cell cycle regulator Swi6 and cell division control protein 10 (Cdc10) and Notch in *Drosophila melanogaster* [10]. AR domains act as scaffolds to facilitate protein–protein interactions in the cell [11, 12] and are present in many eukaryotic proteins, making this domain potentially the most abundant repeat domain in the eukaryotic proteome [13]. FN3 is a very common constituent of animal proteins [14] and has been proven to be an established scaffold for developing nonantibody binding domains [15], which change the conformation of FN3 and eventually translate into structural changes across the membrane [12]. Fibronectin assembly offers functional differentiation from antibodies [16]. Studies have shown that the last 50 amino acids of PTEN, which match the intronic region between exons 3 and 4 of ANKFN1, are related to human height [17], but their involvement in cancer has never been reported [10, 18].

The RAS-RAF-MEK-ERK pathway is the most well-studied mitogen-activated protein kinase (MAPK) cascade and is critical for cell proliferation, differentiation, and survival. Moreover, uncontrolled cell proliferation is a hallmark of cancer in which cell cycle progression is deregulated [19, 20]. Recently, the MEK/ERK signaling pathway in HCC has gained renewed attention from basic and clinical researchers. This signaling pathway is activated in more than 50% of human HCC cases [21].

Cyclin D1, a member of the highly conserved cyclin family, is encoded by the CCND1 gene in humans and is an important regulator of cyclin-dependent kinases, such as CDK4 and CDK6, to control the G1/S transition of the cell cycle. Cyclin D1 and CDK4/6 play a key role in cell cycle progression by phosphorylating and inactivating retinoblastoma protein [22]. The activities of cyclin D1-CDK4/6 are regulated by diverse mechanisms, such as the MEK/ERK signaling pathway.

In this study, we showed that ANKFN1 was upregulated to different degrees in HCC tissues and cell lines and that high HCC expression correlated with poor prognosis in HCC patients. Furthermore, the knockdown of ANKFN1 significantly attenuated HCC cell proliferation and promoted apoptosis in vitro and in vivo. In addition, ANKFN1 overexpression reversed the above phenomenon. In addition, our results highlighted that ANKFN1 functions as a regulator of HCC progression by regulating the MEK/ERK/c-Myc/cyclin D1/Cdk4/Cdk6 pathway.

## Results

### ANKFN1 is frequently overexpressed and associated with a poor prognosis in patients with HCC

**Fig. 1:**
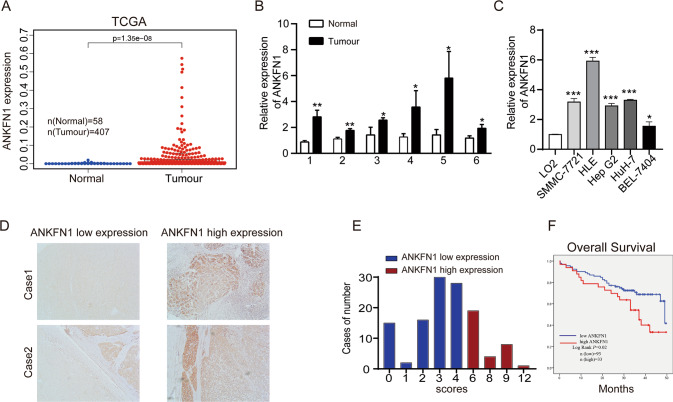
ANKFN1 is frequently overexpressed and associated with a poor prognosis in HCC patients. **A** ANKFN1 was found to be overexpressed in HCC tissues versus normal tissues according to analysis of TCGA HCC data using the R language package “limma”. **B** qRT–PCR analysis of ANKFN1 mRNA expression levels in HCC tissue compared with paracarcinoma tissue. **C** qRT–PCR analysis of ANKFN1 mRNA expression levels in HCC cells (SMMC-7721, HLE, Hep G2, HuH7, BEL-7404) compared with normal liver cells (LO2). **D** Representative images of ANKFN1 IHC staining in HCC tissues of high and low level. Original magnification: ×100. **E** HCC tissues stratified by the IHC staining index. **F** Kaplan–Meier analysis of the OS of 126 HCC patients stratified by ANKFN1 expression. ANKFN1 ankyrin repeat and fibronectin type III domain containing 1, HCC hepatocellular carcinoma, TCGA The Cancer Genome Atlas, qRT‐PCR quantitative real‐time polymerase chain reaction, IHC immunohistochemistry. *P < 0.05; **P < 0.01; ***P < 0.001.

To elucidate the role and clinical significance of ANKFN1 in HCC, we first analyzed ANKFN1 levels using the TCGA database. The results showed that the mean ANKFN1 expression was upregulated in HCC tissues (*n* = 407) compared with noncancerous tissues (*n* = 58) (*P* < 0.001) (Fig. 1A). Therefore, to assess whether ANKFN1 was ectopically overexpressed in HCC, we evaluated ANKFN1 mRNA expression in six freshly frozen paired HCC tissues by qRT–PCR analysis. Consistent with the findings obtained using TCGA, the mRNA levels of ANKFN1 were differentially upregulated in HCC tissues compared with adjacent noncancerous tissues (Fig. 1B). In addition, increased ANKFN1 expression was also detected in five HCC cell lines (SMMC-7721, HLE, Hep G2, HuH-7, and BEL-7404) compared with the normal liver cell line LO2 (Fig. 1C). Thus, we speculated that ANKFN1 may be highly expressed and play important roles in HCC development. To explore the relationship between ANKFN1 expression and the survival of HCC patients, IHC analysis was applied to detect ANKFN1 protein expression levels in 126 tumor tissues. The clinical data of all the patients are shown in Table 1. The results showed that ANKFN1 expression was positively associated with cirrhosis (*P* = 0.005) and serum alpha-fetoprotein (AFP) levels (*P* = 0.045). However, there was no correlation between ANKFN1 expression and other clinicopathological factors, including age, sex, Edmondson’s grade, tumor size (≤5 vs. >5), intrahepatic metastasis, tumor stage (I–II vs. III–IV), or hepatitis B virus (HBV) infection (Table 1). ANKFN1 protein expression was primarily detected in the cytoplasm (Fig. 1D). Kaplan–Meier survival analysis was used to evaluate the effects of ANKFN1 expression on the overall survival (OS) time of patients with HCC. We observed that patients with high ANKFN1 staining had a significantly shorter OS than patients with low ANKFN1 staining (*P* = 0.02) (Fig. 1E, F). Furthermore, the univariate Cox proportional hazard analysis results revealed that Edmondson’s grade (high‐medium vs. low; *P* = 0.039), cirrhosis (*P* = 0.002), and ANKFN1 protein level (low vs. high; *P* = 0.046) were indicators of worse survival in HCC patients. Multivariate Cox proportional hazard analysis showed that the combination of ANKFN1 protein levels (*P* = 0.016) and cirrhosis (*P* = 0.023) was an independent prognostic factor for worse OS in patients with HCC (Table 2). Taken together, these findings suggest that ANKFN1 expression is upregulated in HCC tissues/cells and associated with a poor prognosis in patients with HCC.

**Table 1:** The basic information of 126 patients with HCC.

Clinicopathological features	Number	Low expression, *N* (%)	High expression, *N* (%)	*P* value
Age				
<60	69	49 (71.0)	20 (29.0)	0.66
≥60	57	44 (77.2)	13 (22.8)	
Gender				
Male	103	74 (71.8)	29 (28.2)	0.288
Female	23	19 (82.6)	4 (17.4)	
Tumor size				
≤5 cm	66	46 (69.7)	20 (30.3)	0.314
>5 cm	60	47 (78.3)	13 (21.7)	
Edmondson’s grade				
I–II	37	18 (48.6)	19 (51.4)	0.274
III–V	89	75 (84.3)	14 (15.7)	
Cirrhosis				
Negative	65	55 (84.6)	10(15.4)	0.005*
Positive	61	38 (62.3)	23(37.7)	
AFP(ng/ml)				
≤20	49	41(83.7)	8 (16.3)	0.045*
>20	77	52 (67.5)	25 (32.5)	
HBV				
Negative	13	9 (69.2)	4 (30.8)	0.683
Positive	110	82 (74.5)	28 (25.4)	
HCV	3	2 (66.7)	1 (33.3)	
Intrahepatic metastasis				
Negative	114	83 (72.8)	31 (27.2)	0.434
Positive	12	10 (83.3)	2 (16.7)	

*HCC* hepatocellular carcinoma.

*P < 0.05.

**Table 2:** Univariate and multivariate Cox regression analysis of overall survival in 126 HCC patients.

	Overall survival		
Variables	HR	95% CI	*P* value
Univariate analysis			
Age	0.795	0.457–1.384	0.417
Gender	0.445	0.176–1.120	0.086
Tumor size	1.201	0.692–2.083	0.515
AFP (ng/mL,≤20/>20)	1.381	0.780–2.447	0.268
Cirrhosis	2.429	1.370–4.308	0.002**
Edmondson’s grade	1.578	1.024–2.433	0.039*
HBV	0.436	0.203–0.938	0.034
ANKFN1 expression	1.79	1.009–3.175	0.046*
Multivariate analysis			
AFP (ng/mL,≤20/>20)	1.13	0.610–2.093	0.697
Tumor size	1.094	0.616–1.943	0.76
Cirrhosis	2.047	1.102–3.800	0.023*
Edmondson’s grade	0.716	0.323–1.587	0.411
HBV	0.447	0.197–1.012	0.053
ANKFN1 expression	2.691	1.199–6.039	0.016*

*CI* confidence interval, *HR* hazard ratio, *HCC* hepatocellular carcinoma.

*P < 0.05. **P < 0.01.

### Knockdown of ANKFN1 suppressed HCC cell growth through the induction of G1-S cell cycle arrest and cell apoptosis in vitro

**Fig. 2:**
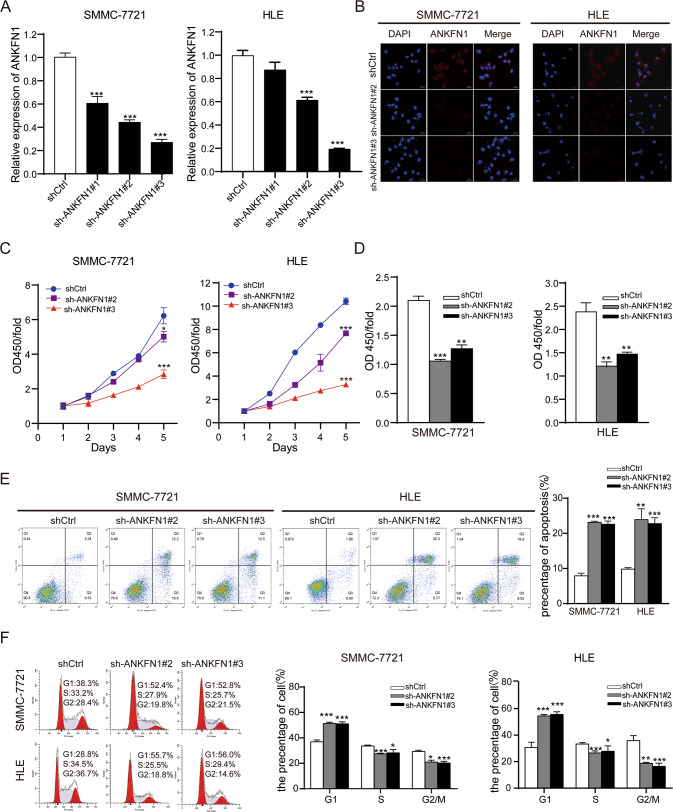
Knockdown of ANKFN1 suppressed HCC cell growth through induction of G1-S cell cycle arrest and cell apoptosis. **A** qRT‐PCR assay analyses of ANKFN1 expression levels in SMMC-7721 and HLE cells infected with sh-ANKFN1-RNA. **B** Immunofluorescence assay analyses of ANKFN1 expression levels in SMMC-7721 and HLE cells infected with sh-ANKFN1-RNA. Scale bars, 50 μm, 20 μm. **C** The effect of ANKFN1 knockdown on SMMC-7721 and HLE cell proliferation was assessed by the CCK-8 assay. **D** The effect of ANKFN1 knockdown on SMMC-7721 and HLE cell proliferation was assessed by the BrdU assay. **E** and **F** Flow cytometry analysis of cell apoptosis and cycle distribution in SMMC-7721 and HLE cells with or without ANKFN1 knockdown. ANKFN1 ankyrin repeat and fibronectin type III domain containing 1, HCC hepatocellular carcinoma, qRT‐PCR quantitative real‐time polymerase chain reaction, IHC immunohistochemistry. *P < 0.05; **P < 0.01; ***P < 0.001.

Increased ANKFN1 expression implies that ANKFN1 may function as an oncogene in the tumorigenesis of HCC. To investigate this possibility, ANKFN1 expression was knocked down in SMMC-7721 and HLE cells with relatively high ANKFN1 expression, as shown in Fig. 1C. The downregulation of ANKFN1 in SMMC-7721 and HLE cells was demonstrated by qRT‐PCR (Fig. 2A) and IF analyses (Fig. 2B). According to the results, we chose sh-ANKFN1#2 (*P* < 0.001) and sh-ANKFN1#3 (*P* < 0.001) for subsequent experiments. Knockdown of ANKFN1 significantly suppressed the proliferation of SMMC-7721 and HLE cells, as determined by CCK-8 (*P* < 0.001) (Fig. 2C) and BrdU (*P* < 0.001) (Fig. 2D) assays. To characterize the mechanism by which ANKFN1 knockdown suppresses HCC cell growth, the effects of ANKFN1 knockdown on the cell cycle and apoptosis were determined by flow cytometry cell cycle distribution and apoptosis assays. There was a significant increase in apoptosis in SMMC-7721 and HLE cells transfected with sh-ANKFN1#2 or sh-ANKFN1#3 compared with control cells (Fig. 2E). Our results showed that the knockdown of ANKFN1 increased the proportion of cells entering G1 phase and decreased the proportion of cells entering S phase, indicating that the knockdown of ANKFN1-induced cell cycle arrest in G1 phase in HCC cells (*P* < 0.001) (Fig. 2F). In summary, ANKFN1 knockdown suppressed HCC cell growth through the induction of G1-S cell cycle arrest and cell apoptosis.

### Knockdown of ANKFN1 inhibits HCC cell migration and invasion in vitro

**Fig. 3:**
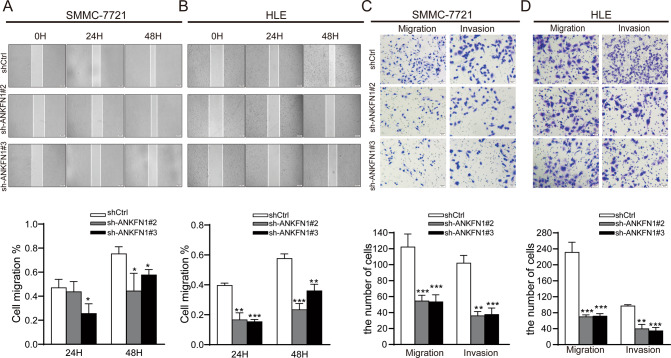
Knockdown of ANKFN1 inhibits HCC cell migration and invasion in vitro. **A** The results of a wound-healing assay showed the effects of ANKFN1 on the cell migration ability of sh‐ANKFN1 #2‐ and sh‐ANKFN1 #3‐infected SMMC-7721 cells. **B** The results of a wound-healing assay showed the effects of ANKFN1 on the cell migration ability of sh‐ANKFN1 #2‐ and sh‐ANKFN1 #3‐infected HLE cells. **C** The effect of ANKFN1 knockdown on SMMC-7721 cell migration and invasion was assessed by a transwell assay. **D** The effect of ANKFN1 knockdown on HLE cell migration and invasion was assessed by a transwell assay. ANKFN1 ankyrin repeat and fibronectin type III domain containing 1, HCC hepatocellular carcinoma. *P < 0.05; **P < 0.01; ***P < 0.001.

**Fig. 4:**
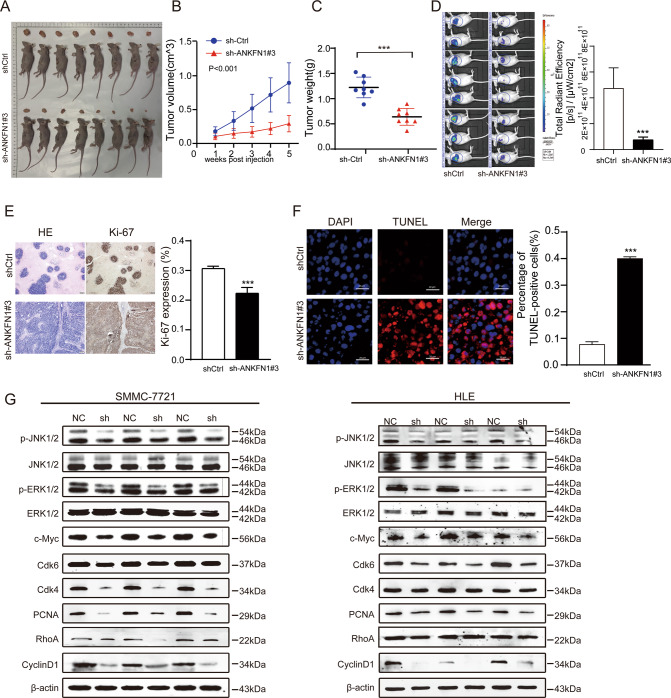
The downregulation of ANKFN1 inhibits HCC growth and promotes apoptosis in nude mice. **A** Representative images of the subcutaneous tumors formed in nude mice between the scramble and ANKFN1-knockdown groups. **B** Statistical comparison of the difference in tumor volume between the scramble and ANKFN1-knockdown groups. **C** Statistical comparison of the difference in tumor weight between the scramble and ANKFN1-knockdown groups. **D** Bioluminescence images of the subcutaneous tumors formed in nude mice between the scramble and ANKFN1-knockdown groups. **E** IHC analysis of HE and Ki-67 expression levels in xenograft tumor tissues in the scramble and ANKFN1-knockdown groups. Scale bars, 200 μm. **F** TUNEL assay for cell apoptosis in tumor tissues developed from SMMC-7721 cells with or without ANKFN1 knockdown. Scale bars, 20 μm. **G** Western blot analysis of the expression of the MAPK signaling pathway proteins p-ERK1/2 and p-JNK1/2 and c-Myc, Cdk6, Cdk4, PCNA, and RhoA in SMMC-7721 and HLE cells with or without ANKFN1 knockdown. ANKFN1 ankyrin repeat and fibronectin type III domain containing 1, HCC hepatocellular carcinoma; *P < 0.05; **P < 0.01; ***P < 0.001.

The effects of ANKFN1 knockdown on the migration and invasion of HCC cells were also explored. Wound-healing and transwell migration assays were performed to examine the effect of ANKFN1 on cell migration. A lower migration rate was observed in SMMC-7721 and HLE cells transfected with sh-ANKFN1#2 and sh-ANKFN1#3 than in control cells. (Fig. 3A–D). In addition, ANKFN1 knockdown also inhibited the invasion abilities of SMMC-7721 and HLE cells, as shown by the transwell Matrigel invasion assay (Fig. 3C, D). Previous studies have shown that RhoA/JNK plays crucial roles during cancer metastasis by decreasing cell–cell contact and increasing cell migration and invasion [23, 24]. To further clarify the role of ANKFN1 in HCC migration and invasion in vitro, we postulated that ANKFN1 promotes cancer migration and invasion by regulating the RhoA/JNK signaling pathway. Our results showed that the knockdown of ANKFN1 decreased RhoA and JNK levels in HCC cells (Fig. 4G).

### The downregulation of ANKFN1 inhibits the growth of HCC and promotes apoptosis in nude mice

To evaluate the function of ANKFN1 in tumorigenesis in vivo, a subcutaneous xenograft mouse model was established. SMMC-7721 cells stably transfected with sh-ANKFN1#3 or shCtrl were injected into the flanks of nude mice to construct xenograft models. Representative images of the subcutaneous tumors formed in nude mice between the scramble and ANKFN1 knockdown groups are shown in Fig. 4A. We observed that the tumor volumes and weights in the sh-ANKFN1 #3 group were lower than those in the scramble group (*P* < 0.001) (Fig. 4B, C). Representative bioluminescence images were captured of the subcutaneous tumors that formed in nude mice in the scramble and ANKFN1-knockdown groups. The total fluorescence of the tumors in the ANKFN1-knockdown group was significantly lower than that in the control group (Fig. 4D). In addition, an IHC assay was performed to detect the level of Ki-67 protein expression, which is closely associated with cell proliferation. As shown in Fig. 4E, in line with the in vitro results, significantly fewer proliferating cells and more apoptotic cells were detected in xenografts from the sh-ANKFN1#3 group than in those from the shCtrl group, as determined by Ki-67 and TUNEL staining assays, respectively (Fig. 4E, F).

### Overexpression of ANKFN1 promotes HCC cell growth and suppresses HCC cell apoptosis in vitro and in vivo

**Fig. 5:**
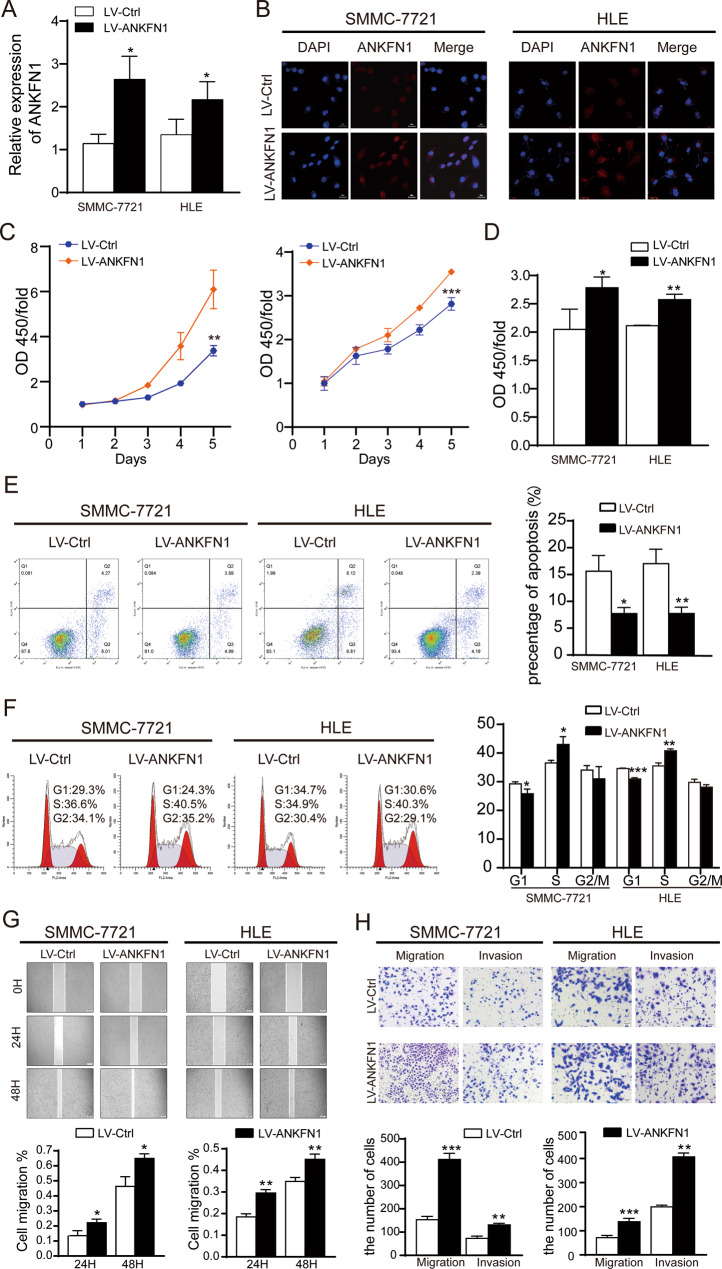
Overexpression of ANKFN1 promotes HCC cell growth, migration, and invasion in vitro. **A** qRT‐PCR assay analyses of ANKFN1 overexpression levels in SMMC-7721 and HLE cells infected with or without LV-ANKFN1. **B** Immunofluorescence assay analyses of ANKFN1 overexpression levels in SMMC-7721 and HLE cells infected with or without LV-ANKFN1. **C** The effect of ANKFN1 overexpression on SMMC-7721 and HLE cell proliferation was assessed by the CCK-8 assay. **D** The effect of ANKFN1 overexpression on SMMC-7721 and HLE cell proliferation was assessed by the BrdU assay. **E** The effect of ANKFN1 overexpression on the apoptosis of SMMC-7721 and HLE cells was analyzed by flow cytometry. **F** The cell cycle distribution of ANKFN1-overexpressing SMMC-7721 and HLE cells was analyzed by flow cytometry. **G** The results of a wound-healing assay showed the effects of ANKFN1 on the cell migration ability of LV-Ctrl- and LV-ANKFN1-infected SMMC-7721 and HLE cells. **H** The effect of ANKFN1 overexpression on SMMC-7721 and HLE cell migration and invasion was assessed by a transwell assay. ANKFN1 ankyrin repeat and fibronectin type III domain containing 1, HCC hepatocellular carcinoma; *P < 0.05; **P < 0.01; ***P < 0.001.

To provide further support for the promoting effects of ANKFN1 on cell growth and metastasis in HCC, ANKFN1 was overexpressed in SMMC-7721 and HLE cells. The overexpression of ANKFN1 in SMMC-7721 and HLE cells was validated by qRT‐PCR (Fig. 5A) and IF analyses (Fig. 5B). The upregulation of ANKFN1 markedly increased the proliferation of SMMC-7721 and HLE cells, as determined by CCK-8 (Fig. 5C) and BrdU (Fig. 5D) assays. As shown in Fig. 5E, there was a significant reduction in apoptosis in SMMC-7721 and HLE cells transfected with LV-ANKFN1 compared with control cells. In addition, the forced expression of ANKFN1 obviously enhanced the proportion of cells in the S phase of the cell cycle in HCC cells (Fig. 5F).

**Fig. 6:**
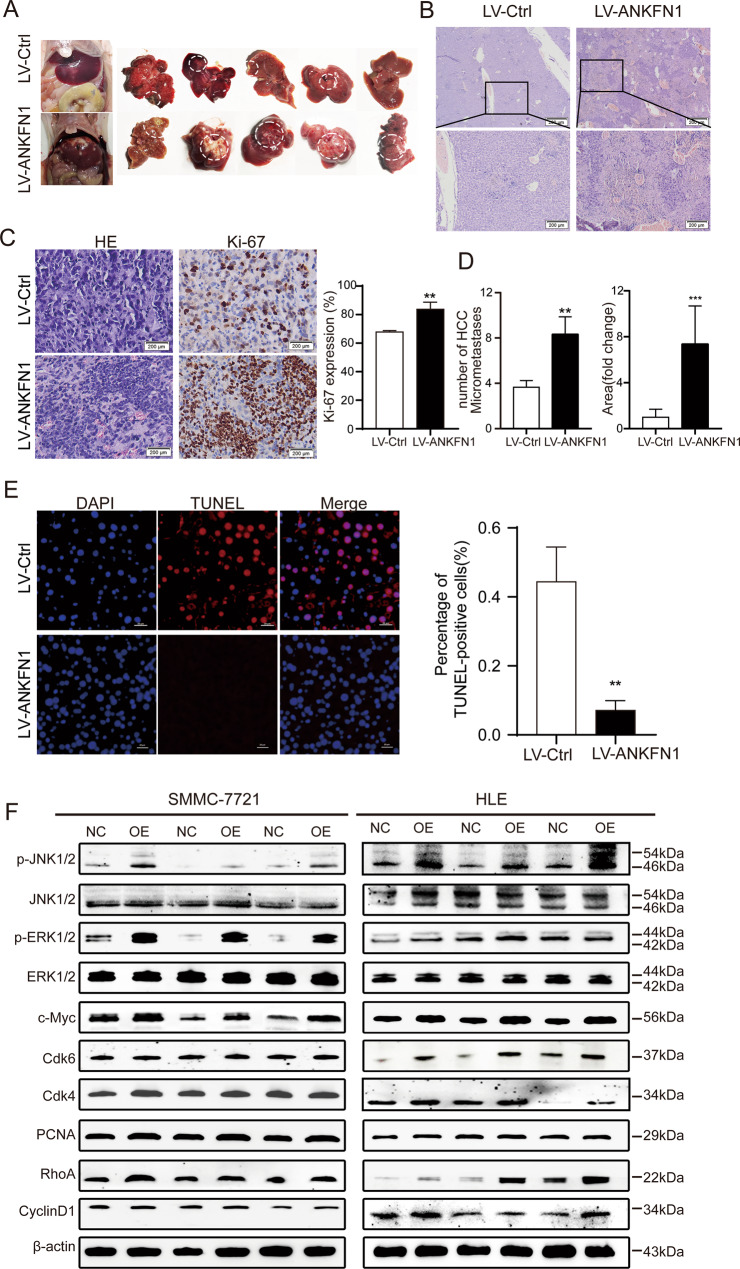
Overexpression of ANKFN1 promotes HCC migration and metastasis in vivo. **A** Liver tissues from animals with tumor xenografts inoculated with SMMC-7721 cell lines stably expressing ANKFN1. **B** HE staining of orthotopic liver transplantation tumors. **C** IHC analysis of HE and Ki-67 expression levels in xenograft tumor tissues in the scramble and ANKFN1-overexpression groups. Scale bars, 200 μm. **D** Statistical graph of the number and area of liver metastases in nude mice. **E** TUNEL assay for cell apoptosis in tumor tissues developed from SMMC-7721 cells with or without ANKFN1 overexpression. Scale bars, 20 μm. **F** Western blot analysis of the expression of the MAPK signaling pathway proteins p-ERK1/2 and p-JNK1/2 and c-Myc, Cdk6, Cdk4, PCNA, and RhoA in SMMC-7721 and HLE cells with or without ANKFN1 overexpression. ANKFN1, ankyrin repeat and fibronectin type III domain containing 1, HCC hepatocellular carcinoma; *P < 0.05; **P < 0.01; ***P < 0.001.

Next, we injected ANKFN1-overexpressing cells into the left hepatic lobe of mice with a microsyringe to establish an orthotopic liver tumor model. Similar to the in vitro results, compared with the control conditions, the overexpression of ANKFN1 promoted HCC growth, as indicated by changes in tumor volume (Fig. 6A, D). Furthermore, our results showed fewer apoptotic cells and a higher intensity of Ki-67 staining in murine xenografts from the ANKFN1-overexpressing group than in those from the shCtrl group, as determined by TUNEL staining assays (Fig. 6E) and Ki-67 staining (Fig. 6C), respectively.

### Overexpression of ANKFN1 promotes HCC cell migration and invasion in vitro and in vivo via the RhoA-ROCK-JNK signaling pathway

Cancer cell migration and invasion are crucial events in HCC metastasis. Therefore, we detected the effect of ANKFN1 on HCC cell migration and invasion. The results of xenograft HCC tumor experiments in nude mice showed that the overexpression of ANKFN1 promoted HCC cell migration and invasion. Consistently, the number and area of intrahepatic metastasis nodules were significantly increased in the ANKFN1-overexpressing group compared with the control group (Fig. 6B, D). Similarly, we conducted western blot analysis to evaluate the expression of RhoA/JNK signaling pathway proteins, as shown in Figs. 6F and S2. Taken together, these findings suggest that ANKFN1 promotes HCC metastasis by regulating the RhoA/JNK signaling pathway.

### ANKFN1 promotes HCC progression through the MEK-ERK-cyclin D1 and RhoA-ROCK-JNK pathways

Cancer is often described as a disease of cell proliferation. However, few targeted therapies are aimed against the specific regulatory machinery that drives entry and progression throughout the cell division cycle [25]. The MAPK signaling cascade is a highly evolutionarily conserved signaling pathway that participates in a myriad of cellular functions, including cell survival, proliferation, growth, and apoptosis [26, 27]. As shown in previous results, ANKFN1 can promote HCC cell proliferation. Cell cycle proteins that are often dysregulated in malignant cells, such as cyclin-dependent kinase (Cdk) 4 and Cdk6, have attracted considerable interest as potential targets for cancer therapy [28]. Therefore, we evaluated the effects of ANKFN1 on the phosphorylation levels of ERK. To further explore whether ANKFN1 affects cell cycle proteins to influence HCC cell proliferation, we assessed the levels of cell cycle-related molecules, such as cyclin D1, Cdk4, Cdk6, and proliferating cell nuclear antigen (PCNA). Western blot analyses showed that ANKFN1 knockdown decreased the levels of phosphorylated ERK without altering the total ERK levels (*P* < 0.05) (Figs. 4G, S1). The expression levels of c-Myc, cyclin D1, CDK4, CDK6 and PCNA were also reduced in SMMC-7721/HLE cells stably transfected with sh-ANKFN1#2 or sh-ANKFN1#3 compared with control cells (Figs. 4G, S1). Consistently, the downregulation of ANKFN1 sharply reduced the tumor volume (Fig. 4B) and nude mouse weight (Fig. 4C). The results indicated that inactivated ANKFN1 blocked the MEK/ERK signaling pathway. The MEK/ERK signaling pathway regulates cell functions, including proliferation, cell survival, and apoptosis [29, 30], in keeping with Figs. 2C–F and 3A–D. All of these results suggest that ANKFN1 knockdown inhibits proliferation and induces apoptosis in HCC cells by suppressing MEK/ERK/c-Myc to regulate the cyclin D1/Cdk4/6 signaling pathway.

As expected, the Western blot analyses revealed that cyclin D1/Cdk4/6 and MEK/ERK signaling pathway proteins (p-ERK, c-Myc, cyclin D1, Cdk4, Cdk6, and PCNA) were more strongly expressed in SMMC-7721 and HLE cells transfected with LV-ANKFN1 than in control cells (Figs. 6F, S2), but the results were reversed in ANKFN1-knockdown SMMC-7721 and HLE cells (Figs. 4G, S1). Collectively, these results demonstrate that ANKFN1 can promote HCC cell proliferation and block HCC cell apoptosis in vitro and in vivo.

**Fig. 7:**
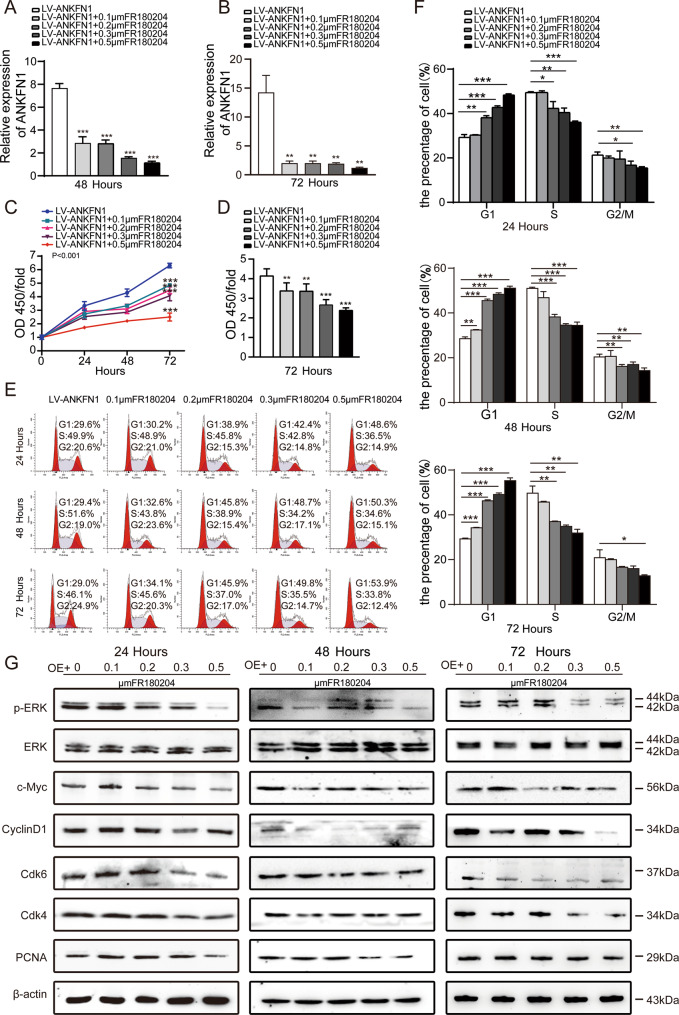
ANKFN1 downregulation is mainly mediated by the downregulation of p-ERK in HCC cells. **A** and **B** qRT–PCR analyses of the expression of ANKFN1 in LV-ANKFN1-SMMC-7721 cells after induction with the ERK inhibitor FR180204 at 48 h and 72 h. **C** The effect of FR180204 on SMMC-7721 cells infected with LV-ANKFN1 cell proliferation was assessed by the CCK-8 assay at 24 h, 48 h, and 72 h. **D** The effect of FR180204 on SMMC-7721 cells infected with LV-ANKFN1 cell proliferation was assessed by the BrdU assay at 72 h. **E** and **F** Flow cytometry analysis of the cell cycle distribution in SMMC-7721 cells infected with LV-ANKFN1 and induced with the ERK inhibitor FR180204 at 24 h, 48 h, and 72 h. **G** Western blot analysis of p-ERK, c-Myc, cyclin D1, Cdk4, Cdk6, and PCNA levels in SMMC-7721 cells infected with LV-ANKFN1 and induced with the ERK inhibitor FR180204 at 24 h, 48 h, and 72 h. *P < 0.05; **P < 0.01; ***P < 0.001.

We further investigated the mechanism of ANKFN1 in HCC cell proliferation. We used the p-ERK inhibitor FR180204 to restrain p-ERK expression in ANKFN1-overexpressing HCC cells. As shown in Fig. 7A, B, ANKFN1 was expressed at low levels in SMMC-7721 cells transfected with LV-ANKFN1 and treated with FR180204 at 0.1 μΜ, 0.2 μΜ, 0.3 μΜ, and 0.5 μΜ at both 48 h and 72 h and showed a decreasing concentration dependence. We then tested whether inhibiting the ERK1/2 signaling pathway at different concentrations can affect ANKFN1-mediated HCC proliferation, growth, and apoptosis at different times by CCK-8 (Fig. 7C), BrdU (Fig. 7D), and flow cytometry cell cycle distribution assays (Fig. 7E, F). Western blot analysis was used to evaluate the expression of proteins in the MEK/ERK/c-Myc and cyclin D1/Cdk4/Cdk6 signaling pathways. As shown in Figs. 7G and S3, at 24 h, 48 h or 72 h, the p-ERK inhibitor FR180204 decreased p-ERK expression. Furthermore, low p-ERK expression reversed the high levels of ANKFN1 (Fig. 7A), c-Myc, cyclin D1, Cdk4, Cdk6, and PCNA (Fig. 7G) in SMMC-7721 cells transfected with LV-ANKFN1. Thus, all of these results indicate that ANKFN1 promotes HCC cell proliferation and apoptosis via the MEK/ERK/c-Myc/cyclin D1/Cdk4/Cdk6 pathway.

## Discussion

HCC is now a global public health challenge, ranking fifth in incidence among cancers worldwide [1], and HCC accounts for 75–85% of primary liver cancers. Its high mortality and recurrence rates result from uncontrolled cancer cell growth and metastasis [31, 32]. The molecular changes and mechanisms involved in HCC tumorigenesis need to be identified. In this study, we identified an ANKFN1/ERK/c-Myc/cyclin D1 regulatory axis in HCC cells, in which ANKFN1 promotes HCC cell proliferation by directly or indirectly positively regulating ERK expression; ERK may activate cyclin D1 and Cdk4/6 expression via c-Myc. Our results elucidate a novel regulatory pathway in HCC cell proliferation, and ANKFN1 may be a potential novel target and molecular diagnostic marker for the treatment of human HCC. We revealed that ANKFN1 expression was frequently upregulated in HCC tissues and cells and was an independent predictive index for OS (Table 2). Furthermore, ANKFN1 expression promoted proliferation and metastasis and inhibited apoptosis in HCC in vitro and in vivo by activating the MEK/ERK/c-Myc/cyclin D1/Cdk4/Cdk6 signaling pathway. In addition, the knockdown of ANKFN1 contrasted with the biological function above. Thus, our research shows for the first time that ANKFN1 acts as an oncogene in HCC and is related to worse prognosis. ANKFN1 may be a potential novel target and molecular diagnostic marker for the treatment of human HCC.

ANKFN1 is a coding protein composed of an AR domain and fibronectin type III domain[33]; previous studies have shown that this gene is related to human height [34, 35], and little has been reported about the function of the gene. In this study, through bioinformatic analysis, we identified the target gene ANKFN1 and speculated that it may be overexpressed in HCC (Fig. 1A). Using molecular biology experiments, we confirmed that ANKFN1 was overexpressed in HCC tissues and cell lines (Fig. 1B, C). Next, through analysis of the ANKFN1 IHC scores in HCC tissues, we observed that high HCC expression correlated with cirrhosis and higher AFP levels (Table 1). Moreover, high ANKFN1 expression was often associated with a poor OS rate in HCC patients (Fig. 1E, F), which was not associated with intrahepatic metastasis or HBV infection because the number of patients with intrahepatic metastasis or HBV infection was small (Table 1). These results confirmed that ANKFN1 may play an important role in HCC tumorigenesis and progression. Further multivariate Cox regression analysis showed that the ANKFN1 protein level, together with cirrhosis, was an independent prognostic factor for OS in patients with HCC (Table 2). These results suggest that ANKFN1 could be a promising prognostic biomarker for HCC patients. To explore the roles of ANKFN1 in HCC tumorigenesis and progression, we conducted a series of cell functional assays. Knockdown of ANKFN1 inhibited HCC proliferation and promoted apoptosis in vitro and in vivo (Figs. 2, 3, 4A–F), and we found that p-ERK, c-Myc, cyclin D1, Cdk4/6 and PCNA were downregulated in sh-ANKFN1#3-transfected cells (Fig. 4G). Conversely, overexpression of ANKFN1 regulated the ERK/c-Myc/cyclin D1/Cdk4/6 pathway (Figs. 6F, S2) and promoted HCC proliferation, metastasis, invasion and repressed apoptosis in vitro and in vivo (Figs. 5, 6A–E). Overall, ANKFN1 is a potential precision cancer treatment target.

MAPK cascades play a central role in human cancer and are hyperactivated in a large variety of tumors [36, 37]. At the end of the MAPK cascade, ERK kinases translocate to the nucleus and phosphorylate a large spectrum of substrates, mostly transcription factors that are involved in a variety of processes, such as proliferation, survival, and differentiation, in a highly context-dependent manner [38, 39]. It has been reported that the extracellular signal-regulated kinases ERK-1 and ERK-2 are evolutionarily conserved, ubiquitous serine-threonine kinases that regulate cellular signaling under both normal and pathological conditions [40]. Moreover, emerging evidence has also shown that sustained activation of the ERK-1/-2 is implicated in the induction of cell death and cell cycle evolve [41, 42] and plays a pivotal role in the cell signal transduction network [43–45]. In this study, we found that the knockdown of ANKFN1 suppressed HCC cell proliferation but promoted apoptosis. ANKFN1 overexpression induced HCC cell growth and inhibited apoptosis. In addition, we revealed that inhibiting ERK expression regulated the proliferation and apoptosis of HCC cells and affected the protein levels of p-ERK, c-Myc, cyclin D1, Cdk4/6, and ANKFN1 in HCC cells (Fig. 7). Therefore, ANKFN1 may mediate the regulation of HCC proliferation and apoptosis via the ERK/c-Myc/cyclin D1/Cdk4/Cdk6 pathway.

RhoA, a Ras homolog gene family member, is a small GTPase protein in the Rho family (P). In humans, RhoA is encoded by the gene *RHOA*, is located on chromosome 3 and consists of an effector domain, four exons, a hypervariable region and a CAAX box motif [46, 47]. Rho-associated kinase (ROCK), a RhoA effector protein, has two ROCK isoforms, ROCK1 and ROCK2, that play crucial roles in various cellular functions, such as cell contraction, migration and actin organization [48, 49]. The JNK/C-JUN pathway regulates cell migration and invasion [50, 51]. Therefore, we assessed the effects of RhoA/ROCK on the JNK pathway. The results showed that downregulation of ANKFN1 control the migration and invasion of HCC cells (Fig. 3) and reduced the levels of RhoA and p-JNK expression (Fig. 4G); in addition, the overexpression of ANKFN1 promoted HCC migration and invasion in vitro and in vivo (Figs. 5G–H, 6B, D) and was related to high expression of RhoA and p-JNK (Figs. 6F, S2). A study suggested that RhoA/ROCK may be related to cyclin D1 levels in ovarian cancer [52], and our results also indicate that the level of RhoA may be affiliated with cyclin D1. In summary, we demonstrated that ANKFN1 can regulate HCC migration and invasion directly or indirectly via the RhoA/ROCK/JNK pathway.

**Fig. 8:**
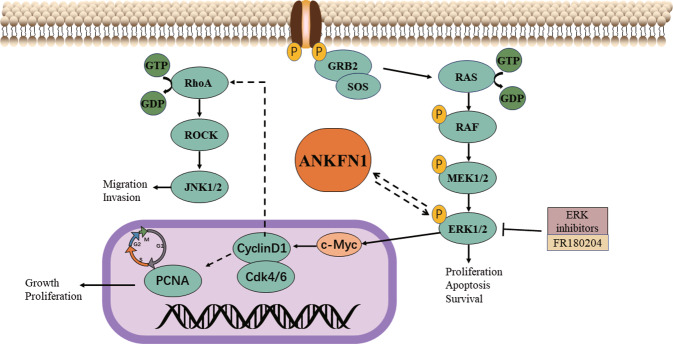
Diagram of the mechanism of the ANKFN1 gene in HCC. ANKFN1 expression promoted proliferation and metastasis and inhibited apoptosis in HCC in vitro and in vivo by activating the MEK/ERK/c-Myc/cyclin D1/Cdk4/Cdk6 signaling pathway. ANKFN1 can regulate HCC migration and invasion directly or indirectly via the RhoA/ROCK/JNK pathway.

In conclusion, our findings demonstrated that ANKFN1 promotes HCC cell proliferation and induces apoptosis via the ERK/c-Myc/cyclin D1/Cdk4/Cdk6 pathway (Fig. 8) and that inhibition of ERK can reverse the biological effects of ANKFN1 in HCC cell lines (Fig. 7). In addition, ANKFN1 can regulate HCC migration and invasion via RhoA/ROCK/JNK. Overall, this paper highlights a novel target gene in HCC and provides valuable information for HCC prognosis. Further insights into the function of ANKFN1 may contribute to the discovery of a promising therapeutic target for improved clinical management of HCC.

## Methods

### Cell lines and cell culture

Three HCC cell lines (SMMC-7721, LO2, Hep G2) were obtained from the Cell Bank of the Institute of Biochemistry and Cell Biology, China Academy of Sciences (Shanghai, China). The BEL-7404, HLE, and Huh-7 cell lines were obtained from the Liver Cancer Institute, Zhongshan Hospital of Fudan University (Shanghai, China). The HLE cell lines used in this study were cultured in Dulbecco’s modified Eagle’s medium (DMEM), and the other cells were cultured in RPMI 1640 medium (Gibco Laboratories, Grand Island, NY, USA). All of the abovementioned cells were cultured in medium containing 10% fetal bovine serum (FBS) (Gibco, New Zealand), 100 U/ml penicillin and 100 µg/ml streptomycin at 37 °C in a 5% CO_2_ incubator. All cell lines were authenticated and characterized by the supplier. Cells were used within 6 months of resuscitation. These cell lines were confirmed to be mycoplasma-free and routinely authenticated by quality examinations of morphology and growth profile.

### Patients and tissue samples

HCC tissues were obtained from 126 patients with HCC who underwent surgery at the First Affiliated Hospital of Bengbu Medical College Hospital (China) from 2017 to 2018. This study was approved by the Ethics Committee of the First Affiliated Hospital of Bengbu Medical College [2021] No. 257.

### Lentiviral vector construction and cell transfection

Lentiviruses containing ANKFN1 short hairpin RNA (sh-ANKFN1) and ANKFN1 overexpression constructs were produced by GeneChem (Shanghai, China). Cells were transfected following the manufacturer’s protocol. The shRNA sequences were as follows: sh-ANKFN1 #1, GCTCTCTGAGGGCATTTATAC; sh-ANKFN1#2, GCCTCTCCCATGTCAGAAATA; and sh-ANKFN1#3, GCATTTATACACAGCACCTGT. The efficiency of gene silencing or overexpression was verified by quantitative real‐time reverse transcription-polymerase chain reaction (qRT‐PCR) assays and immunofluorescence assays.

### Immunohistochemistry (IHC)

All tissue samples were fixed in 4% paraformaldehyde, embedded in paraffin, sectioned at 4-μm thickness and adhered to slides. The tissue microarray slides were deparaffinized with xylene for 30 min and then rehydrated through a graded ethanol series (100%, 95%, 85%, and 75%). Next, antigen retrieval was performed using citric acid buffer (pH 7.8, 0.1 M) for 24 min at approximately 82 °C. The slides were uniformly covered with endogenous peroxidase blocking solution (Solarbio, Beijing, China) for 15 min at room temperature to block the activity of endogenous peroxidase and then blocked with normal goat serum for 15 min. Then, the slides were incubated with anti-ANKFN1 antibody (Abcam, Cambridge, UK) or Ki-67 antibody (Cell Signaling Technology, Danvers, MA, USA) at 4 °C overnight, gently washed with PBS, and incubated with a secondary antibody and streptavidin‐horseradish peroxidase (HRP) for 30 min. Color development was performed using 3,3′‐diaminobenzidine, and counterstaining was performed using hematoxylin. The IHC scores were evaluated independently by two experienced pathologists who were unaware of the pathological grade information. The IHC evaluation scores of each sample were based on the product of the staining intensity score and the extent of the staining area. The staining intensity of ANKFN1 expression was quantified as follows: 0, no staining; 1, mild staining; 2, moderate staining; and 3, intense staining. The extent of ANKFN1 expression was scored as follows: 0, no staining; 1, 1–25%; 2, 26–50%; 3, 51–75%; and 4, 76–100%. Samples were defined as having high ANKFN1 expression when their evaluation scores were greater than 6.

### Immunofluorescence (IF)

The transfected HCC cells were fixed with 4% paraformaldehyde for 20 min and then permeabilized with 0.1% Triton X‐100 for 10 min. After being washed with PBS, the slides were incubated with primary antibodies against ANKFN1 (Abcam, Cambridge, UK) in blocking solution overnight at 4 °C in a humidified chamber. Subsequently, the cells were incubated with fluorescent secondary antibodies for 1 h and then with 4,6‐diamino‐2‐phenylindole for 5 min (DAPI) in blocking solution for 30 min at 37 °C in a humidified chamber. IF signals were visualized by fluorescence microscopy.

### RNA isolation and real-time quantitative PCR (qRT-PCR)

Total RNA was isolated using TRIzol reagent, and the extracted RNA was reverse transcribed into cDNA with the PrimeScript™ 1st Strand cDNA Synthesis Kit. SYBR Green Real-Time PCR Master Mix was used for qPCR detection, and the qPCR program was as follows: step 1, denaturation at 95 °C for 3 min; and step 2, 95 °C for 5 s and 60 °C for 30 s for 40 cycles. A LightCycler 96 Real-Time PCR Detection System (Roche, USA) was used for PCR detection. The primer sequences used in each reaction were as follows: ANKFN1 forward, GGATTCAAAGGGAGTGTACGAC and reverse, CCCGTAAAGAAACGCACCTTC; and GAPDH forward, TGACTTCAACAGCGACACCCA and reverse, CACCCTGTTGCTGTAGCCAAA. The primers were synthesized and purified by Shenggong Biotech (Shanghai, China).

### Cell proliferation assay

The effect of ANKFN1 on HCC cell proliferation was assessed using the Cell Counting Kit-8 (CCK-8) (MedChemExpress, NJ, USA) assay and BrdU assays. For the CCK-8 assay, HLE and SMMC-7721 cells (2000 cells per well) infected with sh-ANKFN1#2, sh-ANKFN1#3, or ANKFN1 overexpression lentivirus (LV-ANKFN1) were seeded into 96‐well plates. After incubating for 1, 2, 3, and 4 days, 10 μl of the CCK‐8 assay solution was added to each well at the indicated times, and the cells were cultured for 1 h in the incubator. The optical densities at 450 nm were measured using an automatic microplate reader (BioTek Instruments Inc., VT, USA). For the BrdU assay, we used a BrdU kit (Beyotime Institute of Biotechnology, Shanghai, China) 72 h after transfection according to the manufacturer’s protocol. The assay was conducted in triplicate, and the absorbance was detected at a wavelength of 450 nm with a microplate reader.

### Wound-healing assay

Transfected HCC cells (2 × 10^5^ cells per well) were seeded into six-well plates and cultured to 90% confluence as a monolayer. Then, a scratch in the monolayer of cells was generated using a 100‐µl plastic pipette tip, and the dislodged cells were removed. Then, the cells were replenished with fresh medium at low serum concentrations or in the absence of serum. Images were captured with a light Olympus microscope at 0, 24, and 48 h. ImageJ software was used to determine the relative migration in each group.

### Transwell assay

For the migration and invasion assays, cells were seeded in the upper chamber of a transwell (8-μm pore size) or in a Matrigel-coated transwell (Corning, NY, USA) in serum-free media. The lower chamber contained 600 μl of basal medium with 10% fetal bovine serum as a chemoattractant. After 48 h of incubation, the nonmigrated or noninvaded cells were gently removed from the upper chamber using a cotton swab. The remaining cells in the lower chamber were fixed with methyl alcohol, stained with a 0.1% crystal violet solution for 15 min and then imaged via microscopy (Olympus Corporation, Tokyo, Japan). For each sample, five random fields (×100 magnification) were selected, and the cells in each field of view were counted.

### Western blot assay

RIPA buffer, protease inhibitors and phosphatase inhibitors were used for total cell protein extraction, and the cells were boiled for 10 min. The protein concentration was determined using a BCA protein assay kit (Thermo Fisher Scientific, Grand Island, NY, USA) according to the manufacturer’s instructions. A PowerPac HV High-Voltage Power Supply (Bio-Rad Laboratories, USA) was used for protein electrophoresis. The samples were then separated by 10% or 12% sodium dodecyl sulfate (SDS)-polyacrylamide gels and transferred onto nitrocellulose membranes. Next, blocking with 5% nonfat milk was performed for 1 h at room temperature. The membranes were washed and incubated with primary antibodies against c-Myc, cyclin D1, p-JNK, p-ERK, ERK1 + ERK2, JNK1 + JNK2 + JNK3, PCNA, Cdk4, Cdk6, RhoA, and β‐actin, which were provided by Abcam (Cambridge, UK), at 4 °C overnight. The membranes were then incubated with the appropriate HRP-labeled secondary antibodies for 2 h at room temperature. Finally, a hypersensitive enhanced chemiluminescence (ECL) kit was used to detect specific protein bands using a Bio-Rad ChemiDoc XRS Imaging System (Bio-Rad Laboratories, CA, USA) for visualization and quantification; ImageJ computer software (Software Inquiry; Quebec, Canada) was used to quantify the bands.

### Flow cytometry analysis of the cell cycle and cell apoptosis

For cell cycle analysis, cells were plated in a 6‐well culture plate and grown for 24 h. Next, the cells were trypsinized, washed twice with cold PBS and fixed with cold 70% ethanol at −20 °C overnight. The cells were then washed twice with PBS and incubated with 10 mg/ml RNase A, 400 mg/ml propidium iodide and 0.1% Triton X in PBS at room temperature (RT) for 30 min. The cells were subsequently analyzed by flow cytometry. For cell apoptosis, an annexin V-FITC/PI apoptosis detection kit was used to detect cell apoptosis. HCC cell lines (HLE, SMMC-7721) were treated with unrelated shRNA lentivirus and sh-ANKFN1#2 and sh-ANKFN1#3 lentivirus, and the cells were seeded at a density of 5 × 10^5^ cells per well into 12-well plates and allowed to grow overnight. Then, the cells were harvested, washed, seeded at a density of 5 × 10^5^ cells and allowed to grow overnight. Then, the cells were harvested and washed twice with cold PBS. Next, the cells were resuspended in 400 μl of Annexin-binding buffer before adding 5 µl of annexin V-FITC and 10 µl PI for incubation at room temperature for 25 min. Cell apoptosis was detected using flow cytometry (LSRFortessaTM X-20; BD Biosciences, San Jose, NJ, USA).

### Xenograft mouse models

The mice were maintained in laminar flow cabinets in a specific-pathogen‐free animal laboratory with an artificial 10-h light and 14-h dark cycle. The room temperature was 27 ± 2 °C, and the humidity was 40–60%. Tumor volumes and the weights of the nude mice were recorded every 3 days for up to 28 days. To establish a subcutaneous xenograft model, 1 × 10^6^ SMMC-7721 cells stably infected with scramble or sh-ANKFN1 were subcutaneously injected into the flank of each mouse. The mice were sacrificed 4 weeks after injection. The tumors were weighed, and the volumes were calculated using the following formula: volume = L × W 2 ×0.5 (L, length; W, width). For in vivo growth and metastasis assays, six- to eight-week-old male nude mice orthotopically inoculated into the left hepatic lobe with a microsyringe through an 8-mm transverse incision in the upper abdomen under anesthesia. A total of 1 × 10^6^ cells suspended in 40 μl of a mixture of serum-free 1640/Matrigel (1:1 volume) (BD Biosciences, MA, USA) were injected into each nude mouse. Six or 10 weeks later, the mice were sacrificed, and the tumors and individual liver and lung tissues were excised and fixed with 4% phosphate-buffered neutral formalin for at least 72 h. Metastatic tissues were analyzed by H&E and Ki-67 staining. In addition, the liver tissues were analyzed by TUNEL assay. All animal use procedures were in accordance with the Guide for the Care and Use of Laboratory Animals (NIH publications no. 80-23, revised 1996) and were performed according to the institutional ethical guidelines for animal experiments. Certificate number [2021] No. 313 was used for the animal experiments.

### Bioinformatics analysis

We downloaded HCC data (normal = 58, cancer = 407) from The Cancer Genome Atlas (TCGA) database (https://www.cancer.gov/) and extracted the expression of ANKFN1 in HCC using the R language package “limma”. Using these data, we analyzed the differential expression of ANKFN1 in HCC compared to normal tissues.

### Statistical analysis

The data are presented as the mean ± standard deviation (SD). All statistical analyses were performed using SPSS, version 23.0 (IBM Corp, Armonk, NY, USA). Pearson’s *χ*^2^ test was used to analyze the relationship between ANKFN1 expression and clinicopathological characteristics. A survival curve was constructed, and differences among the groups were calculated using the Kaplan–Meier method and log-rank test. Survival data were investigated using univariate and multivariate Cox proportional hazards regression analyses. *P* < 0.05 was considered significant.

## Supplementary information

supplement figure.1

supplement figure.2

supplement figure.3

Legend of supplementary diagram
