# Supplementary material for: ANKFN1 plays both protumorigenic and metastatic roles in hepatocellular carcinoma
Source: Oncogene. 2022 Jun 21;41(29):3680–93. doi: 10.1038/s41388-022-02380-0 (PMC9287179; doi:10.1038/s41388-022-02380-0)
Supplement: Supplementary file 4 — Legend of supplementary diagram [file 41388_2022_2380_MOESM4_ESM.docx]

Supplement figure 1. The statistical chart of Western Blot in SMMC-7721 and HLE cells infected with sh-ANKFN1#2 or sh-ANKFN1#3 compared with shCtrl group about p-ERK, p-JNK, c-Myc, CyclinD1, Cdk4, Cdk6, PCNA, RhoA. **P* <0.05; ***P* <0.01; ****P* <0.001.

Supplement figure 2. The statistical chart of Western Blot in SMMC-7721 and HLE cells infected with LV-ANKFN1 compared with LV-Ctrl group about p-ERK, p-JNK, c-Myc, CyclinD1, Cdk4, Cdk6, PCNA, RhoA. **P* <0.05; ***P* <0.01; ****P* <0.001.

Supplement figure 3. The statistical chart of Western blot analysis p-ERK, c-Myc, CyclinD1, Cdk4, Cdk6 and PCNA level in SMMC-7721 cells infected with LV-ANKFN1 and induce with ERK inhibitor FR180204 at 24 hour, 48 hour and 72 hour in different concentration. **P* <0.05; ***P* <0.01; ****P* <0.001.
